# A benefit–risk analysis of rotavirus vaccination, France, 2015

**DOI:** 10.2807/1560-7917.ES.2017.22.50.17-00041

**Published:** 2017-12-14

**Authors:** Adnane Lamrani, Pascale Tubert-Bitter, Catherine Hill, Sylvie Escolano

**Affiliations:** 1 Biostatistics, Biomathematics, Pharmacoepidemiology and Infectious Diseases (B2PHI), Inserm, UVSQ, Institut Pasteur, Université Paris-Saclay, Villejuif, France; 2 Centre de Recherche en Epidémiologie et Santé des Population (CESP), Inserm, Villejuif, France

**Keywords:** rotavirus, vaccination, intussusception, risk-benefit analysis, simulations

## Abstract

Two vaccines available for protection against rotavirus gastroenteritis (RVGE), Rotarix and RotaTeq, have contributed to a large decrease in the incidence of paediatric diarrhoea in countries where they have been used. However, they have also led to a small increase in the risk of intussusception. **Methods:** We compare the number of prevented hospitalisations for RVGE to the number of vaccine-induced hospitalised intussusceptions in France. **Results:** With 9.5% coverage (French 2015 estimation), vaccination was estimated to prevent, annually, a median of 1,074 hospitalisations (2.5th and 97.5th percentiles (2.5th–97.5th): 810–1,378) and 1.4 deaths (2.5th–97.5th: 1.2–1.6) from RVGE. It was also estimated to cause, annually, 5.0 hospitalisations (2.5th–97.5th: 3.2–7.7) and 0.005 deaths (2.5th–97.5th: 0.001–0.015) from intussusception. The benefit–risk ratio is therefore 214 (2.5th–97.5th: 128–362) for hospitalisations and 273 (2.5th–97.5th: 89–1,228) for deaths. Under a hypothetical 92% coverage, rotavirus vaccination with Rotarix would avoid 10,459 (2.5th–97.5th: 7,702–13,498) hospitalisations for RVGE and induce 47.0 (2.5th–97.5th: 25.1–81.4) hospitalisations for intussusception annually, thereby preventing 13.7 (2.5th–97.5th: 11.1–15.2) deaths and inducing 0.05 (2.5th–97.5th: 0.01–0.15) deaths. **Conclusion:** The benefit–risk ratio in France is similar to that of other European countries.

## Introduction

Rotavirus is the leading cause of severe gastroenteritis worldwide [1]. This highly infectious pathogen spreads by the faecal-oral route, which means that rotavirus can be spread by contaminated hands, objects, food and water. The incidence of rotavirus gastroenteritis (RVGE) has greatly decreased in all countries where rotavirus vaccination has been included in the national immunisation programme [2]. However, rotavirus vaccines have been shown to increase the risk of intussusception [3-5]. Intussusception is a rare but severe bowel disorder where the bowel telescopes into itself and which is lethal if not treated immediately. Most commonly, it occurs in unvaccinated children between 5 and 10 months of age. The first rotavirus vaccine, Rotashield, was licensed in August 1998 in the United States (US). After reports of its association with an increased risk of intussusception [6], it stopped being recommended in the US a few months later, which led to its withdrawal worldwide. Two vaccines have been marketed and authorised in many countries since then: Rotarix (GlaxoSmithKline Biologicals, North Carolina, US), a monovalent vaccine that is given in two doses to children at 2 and 4 months of age, and RotaTeq (Merck & Co., Pennsylvania, US), a pentavalent vaccine that is given in three doses at 2, 4 and 6 months of age. Both manufacturers recommend that children receive all doses before they turn 8 months of age. With these two new vaccines, some studies have shown an increased risk of intussusception during the first week after the first dose [6-8], and another one has suggested a smaller increase in risk during the second week and third week after the first dose as well as an increased risk after the second dose [9].

As vaccines are administered to healthy individuals, tolerance for risk is low in the general public. Benefit–risk (BR) ratios are estimated to compare vaccine efficacy and vaccine safety. Recent BR studies include precision analysis of BR estimates by taking into account the uncertainty on the parameters [10,11]. Rotavirus vaccination with Rotarix or RotaTeq is an example of where the BR ratio has been assessed in different epidemiological contexts or with different immunisation schedules [12,13].

In low- and middle-income countries, it is not uncommon for infants to die from RVGE: in 2013, the World Health Organization (WHO) estimated that the number of rotavirus deaths in children under 5 years of age was 215,000, with approximately half (49%) occurring in four countries (India, Nigeria, Pakistan, and Democratic Republic of Congo) [14]. BR studies evaluating intussusception risk have been conducted in Mexico, Brazil and Latin America [3,15]. Recently, BR studies have also been conducted in industrialised countries (Australia, England, Japan, US) [9-11,16]. However, the conclusions of a study in one country cannot easily be extrapolated to another because countries have different demographic and epidemiologic characteristics, which are parameters that enter in the BR calculation. In Europe, the vaccine introduction, thus the vaccine coverage, varies greatly, reaching more than 90% in the United Kingdom, Belgium and Finland. Extensive effectiveness research has been conducted in European countries (reviewed in [17]), particularly in those countries that have introduced rotavirus immunisation in national programs [18-20]. The only BR study in a European country was conducted in England [10], were the vaccine is recommended. In France, rotavirus vaccination was recommended by the authorities in November 2013 [21] and was cancelled in April 2015 [22] after a report of two deaths following intussusception in vaccinated infants [23]. Rotarix and RotaTeq are available in France, but neither is reimbursed by the French healthcare system which explains the low coverage.

The aim of this paper is to present a quantitative BR analysis of rotavirus vaccination in France. This analysis will provide useful information to health policymakers in France, a country where skepticism about vaccine safety has been documented in one study as very prevalent [24].

## Methods

### Study design

We conducted a modelling and Monte Carlo simulation study to assess the BR ratio of rotavirus vaccination with Rotarix and RotaTeq in French children under 5 years of age. We used the 2015 population size with respect to the same age group as reference. The benefit of rotavirus vaccinations was defined as the reduction in the number of hospitalisations or deaths from a rotavirus infection due to vaccination and the risk was defined as the increase in number of hospitalisations or deaths from intussusception in that population. The age window of 0 to 5 years, which defines the period of immunisation benefit, is classically used to measure the rotavirus burden. Children are vaccinated during their first year of age and vaccine-induced intussusceptions occur shortly after vaccination, well before 1 year of age.

### Variables

The model comprised three kinds of variables. First, those related to the population of vaccinated children: population size, partial and full vaccination coverage and age at dose administration. Second, those specific to the calculation of benefit, related to RVGE: baseline incidence of hospitalisation for RVGE and its age distribution, vaccine efficacy and mortality because of RVGE. Third, those specific to the calculation of risk, related to intussusception: baseline incidence of hospitalisation for intussusception and its age distribution, vaccine-induced relative risk (RR) and mortality because of intussusception.

### Data sources

#### Population size

According to the National Bureau of Statistics (INSEE), there were 3,967,024 children under 5 years of age in the French population in 2015, including 765,550 children under 1 year of age [25].

#### Rotavirus gastroenteritis

Hospitalisations for RVGE were obtained from the Echantillon Généraliste des Bénéficiaires (EGB), a representative sample (1/97th) from the national database in which both healthcare claims and hospitalisations are recorded [26]. We selected all children under 5 years of age, hospitalised with a diagnosis of rotavirus enteritis (A080 code in the 10th revision of the International Classification of Diseases (ICD10)) occurring between January 2009 and December 2014. The most recent mortality rate in French children under 5 years of age due to RVGE was found in the WHO 2013 report on child rotavirus deaths by country [14].

Vaccine efficacy against RVGE hospitalisations and its decrease with time (waning effect) was modelled using Vezikari's estimates [27-29]. For Rotarix, the efficacy after the first dose was assumed to be constant and equal to 96.0% during the first 6 months after immunisation, and then to decrease linearly between 6 and 12 months after immunisation to reach 90.7% 12 months after immunisation, remaining constant afterwards. After the second dose, the efficacy was assumed to be 99.0% during the first 4 months, and then to decline linearly to reach 92.2% at 10 months, remaining constant afterwards. Corresponding values for RotaTeq are 95.8% and 88.0%, 77.4% and 72.1%, 58.9% and 53.6% for three doses, two doses and one dose, respectively.

We also performed an analysis using a simpler model assuming that efficacy did not decrease after immunisation (no waning effect).

#### Intussusceptions

The number of children under 1 year of age that were hospitalised for intussusception and the age distribution of these children was estimated from the Epistudy registry [30]. This study was undertaken in eastern France, an area recording ca 100,000 annual births, between April 2008 and March 2012. The annual number of births in France 2008–2012 was ca 830,000 [31]. All hospitalised cases that met level 1 of the Brighton collaboration classification and that occurred in one of seven university hospitals in this area were included in this registry. Vaccine-induced risks were those estimated in a paper from Australia [9], a country where both Rotarix and RotaTeq are recommended. In this paper, the risks in the three weeks following each dose of vaccine were estimated. The mortality rate due to intussusception were those estimated in a study reporting deaths observed in seven European countries, not including France [32].

#### Vaccine coverage

Although there was a temporary recommendation between November 2013 and April 2015, no immunisation against rotavirus has been recommended in France since 2015 [21,22]. Furthermore, the two vaccines have never been reimbursed by the French national health plan (Assurance Maladie). The estimation of the coverage was based on 2008 to 2013 sales of a national panel of 3,004 pharmacies across France [33] and was taken to be 9.5% in 2015, 70% with Rotarix and 30% with RotaTeq [34]. Thus, according to this data, fewer than 380,000 of the almost 4 million French children under 5 years of age were vaccinated by one of the two available vaccines. The vaccination schedule and the delay between doses were assumed to be those recommended by the manufacturers, and the weekly number of vaccinated infants was considered to be constant within the recommended age intervals. For Rotarix, the intervals were between 7 and 15 weeks of age for the first dose, and between 11 and 23 weeks of age for the second dose. For RotaTeq, the corresponding age intervals were: 7 to 12 weeks for dose one, 11 to 22 weeks for dose two, and 15 to 32 weeks for dose three.

We also explored the scenario where the rotavirus vaccine would become recommended by the French health authorities, with vaccination coverage reaching 92%. This is the coverage level of the only compulsory vaccine in France, the vaccine against diphtheria-tetanus-poliomyelitis.

Finally, based on Sabbe’s estimation of incomplete vaccination in Belgium [18], we supposed that for Rotarix, 4% of children receiving a first dose did not receive a second one. For RotaTeq, a similar 4% decrease in coverage was applied between the second and first dose and between the third and second dose.

### Statistical methods

#### Benefit calculation

The benefit *B* is defined as the annual number of prevented hospitalizations for RVGE. It depends on the number of infants hospitalised for RVGE at age *w* in the absence of vaccination, noted as *H(w),* on the proportion of the population newly vaccinated by dose *d* at age *w* (*d* = 1,2 for Rotarix, and *d* = 1,2,3 for Rotateq), noted as *v_d_(w)*, and on the efficacy of vaccination at dose *d* on the *t^th^* week after vaccination, noted as *E_d_(t)*. The efficacy is the probability of not being infected once vaccinated, which we assumed did not depend on age of vaccination. The benefit *B* is estimated during the first 5 years of life, i.e. until week 261.

The benefit for Rotarix is:

$$B=\sum_{w=1}^{261}H\left(w\right)\sum_{i=1}^{w}\left[{{\;v_{2}\left(i\right)\;E}_{2}\left(w-i+1\right)\;+\left[v_{1}\left(i\right)-v_{2}\left(i\right)\right]\;E}_{1}\left(w-i+1\right)\right].$$

If the efficacy is constant after vaccination, i.e. in the absence of a waning effect of vaccination, the benefit is simply:

$$B=\sum_{w=1}^{261}H\left(w\right)\left[{{\;V_{2}\left(w\right)\;E}_{2}+\left[V_{1}\left(w\right)-V_{2}\left(w\right)\right]\;E}_{1}\right],$$

where *V_d_(w)* is the vaccine coverage of the population by dose *d* at age *w*, i.e. $V_{d}\left(w\right)=\sum_{i=1}^{w}v_{d}\left(i\right).$

The benefit for RotaTeq, with three doses, is:

$$B=\sum_{w=1}^{261}H(w)\sum_{i=1}^{w}\left[{{\;v}_{3}\left(i\right){\;E}_{3}\left(w-i+1\right)+\left[v_{2}\left(i\right)-v_{3}\left(i\right)\right]\;E_{2}\left(w-i+1\right)+\;\left[v_{1}\left(i\right)-v_{2}\left(i\right)\right]\;E}_{1}\left(w-i+1\right)\right],$$

which, if the vaccine efficacy remains constant after vaccination, reduces to:

$$B=\sum_{w=1}^{261}H\left(w\right)\left[{{\;V_{3}\left(w\right)\;E}_{3}\;+\left[V_{2}\left(w\right)-V_{3}\left(w\right)\right]\;E}_{2}+\;\left[V_{1}\left(w\right)-V_{2}\left(w\right)\right]\;E_{1}\right].$$

The benefit in terms of RVGE-related prevented deaths is the benefit *B* multiplied by the mortality rate of rotavirus infections, which is assumed to be independent of age.

The baseline number *H(w)* was calculated as *H *× *h(w)* where *H* is the annual number of RVGE hospitalisations in infants under 5 years of age and *h(w)* their age distribution, obtained by fitting a Gamma distribution to the observed intussusception data (see under Data sources).

#### Risk calculation

The risk *R* is defined as the annual number of vaccine-induced hospitalisations for intussusception. It depends on the number of infants experiencing the adverse event in the absence of vaccination at age *w*, noted as *A(w)*, on the proportion of the population vaccinated by dose *d* at age *w*, *v_d_(w)*, and on the RR of the adverse event in the *t^th^* week after dose *d* of vaccination, noted as *RR_d_(t)*. For Rotarix and RotaTeq, the risk is significantly increased only in the 3 weeks following dose one and dose two of vaccination [9], hence *t* = 1, 2, 3. The risk *R* is estimated during the first year of life, i.e. until week 52.

The risk is:

$$R=\sum_{w=1}^{52}\left[v_{1}\text{(w)}\sum_{t=1}^{3}A\left(w+t-1\right)\left[{RR}_{1}\left(t\right)-1\right]+v_{2}\text{(w)}\sum_{t=1}^{3}A\left(w+t-1\right)\left[{RR}_{2}\left(t\right)-1\right]\right].$$

For example, risk formulation for Rotarix reduces to:

$$R=\sum_{w=7}^{15}v_{1}\sum_{t=1}^{3}A\left(w+t-1\right)\left[{RR}_{1}\left(t\right)-1\right]+\sum_{w=11}^{23}v_{2}\sum_{t=1}^{3}A\left(w+t-1\right)\left[{RR}_{2}\left(t\right)-1\right],$$

since summed values are zeros for *w* > 7 or *w* > 15 when the first dose is considered, and for *w* > 11 or *w* > 23 when the second dose is considered.

The risk for intussusception-related deaths is the risk *R* multiplied by the case fatality rate of intussusception, which is assumed to be independent of age.

The baseline number *A(w*) was calculated as *A* × *a(w)* where *A* is the annual number of intussusception cases and *a(w)* its age distribution, obtained by fitting a Gamma distribution to the observed intussusception data (see under Data sources).

#### Interval estimation of benefit-risk ratio

All parameters used as inputs to calculate the number of prevented rotavirus events (benefit) and the number of caused intussusception events (risk) were based on published studies or governmental data sources; as estimates themselves, these input values entail uncertainty. Uncertainties were modelled with probability distributions and a large number of simulations were conducted to provide point estimates as well as uncertainty intervals for the BR ratio estimates. Gamma distributions were assumed for the baseline annual rate of hospitalisations for RVGE *(H)* and for intussusception (*A)*, lognormal distributions were assumed for the vaccine-induced relative risks of intussusception *RR_d_(t)*, and Gaussian distributions were assumed for the efficacy *E_d_*. The parameters of these distributions were selected to correspond to published estimates for central values and confidence intervals. The calculations of *B*, *R* and *BR* ratio were iterated 20,000 times, allowing estimation of median and empirical 95% intervals, further referred to as 2.5th and 97.5th percentiles (2.5th–97.5th), for the estimators. To mimic the French context where the two vaccines are used, each iteration was performed as follows: once the baseline numbers of RVGE and intussusception were drawn, the benefit and the risk were calculated from a mixture of vaccinated cases (Rotarix and RotaTeq).

The SAS 9.4 software (SAS Institute Inc. Cary, North Carolina, US) was used to implement the model and perform all simulations.

## Results

### Benefit input parameters

**Figure 1:**
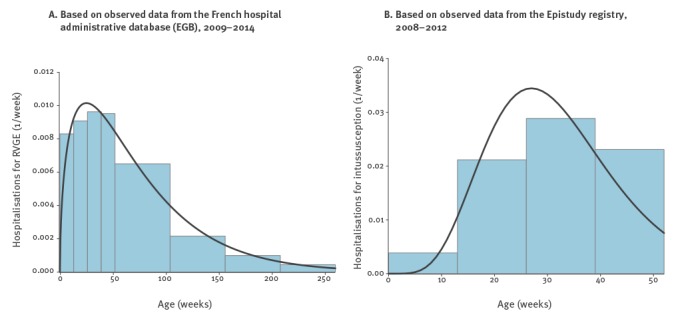
Age distribution of hospitalisations because of rotavirus gastroenteritis (A) and intussusception (B), France

EGB: Echantillon Généraliste des Bénéficiaires; RVGE: rotavirus gastroenteritis.

The annual incidence of RVGE hospitalisations was estimated to be 3.1‰ based on the 703 cases registered between 2009 and 2014 in the EGB. This incidence led to an average number of 12,400 cases of RVGE hospitalisations in infants under 5 years of age annually. The age distribution is displayed in Figure 1 (A).

The solid line in both figures depicts the fitted Gamma distribution.

The mortality rate due to RVGE was 4.0 per 1,000,000, based on the 16 rotavirus deaths reported to the WHO for 2013 [14]. The upper part of Table 1 summarises the chosen distributions and parameters for Rotarix, while the corresponding information for RotaTeq is detailed in the upper part of Table 2.

**Table 1:** Model input parameters for Rotarix and their distributions for interval estimation

Input parameter		Mean (n or %)	(SD) or 95% confidence interval	Reference	Probability distribution
Benefit: prevented hospitalisation for RVGE before 5 years of age	Annual incidence of RVGE hospitalisations per 1,000 infants	3.1	(0.4)	^a^	Gamma shape = 61.2, scale = 5.09e-5
	Number of RVGE deaths per year	16	15–18	[14]	Lognormal μ = ln(16), σ = 0.046
	Efficacy after first dose				
	During first 6 months	96.0%	90.2%–98.8%	[28]	Beta α = 75.64, β = 3.15
	After 12th month	90.7%	85.6%–94.3%		Beta α = 154.4, β = 15.8
	Efficacy after second dose				
	During first 4 months	100.0%	81.8%–100.0%	[28]	Beta α = 1, β = 0.01486
	After 10th month	92.2%	65.6%–99.1%		Beta α = 8.16, β = 0.69
Risk: vaccine-induced hospitalisation for IS before 1 year of age	Annual incidence of IS hospitalisations per 100,000 infants under 1 year of age	28.0	(1.9)	[21]	Gamma shape = 214.4, scale = 1.31e-6
	Case–fatality rate of IS	0.0012	(0.0007)	[32]	Beta α = 3, β = 2584
	Vaccine-induced RR of IS post dose one during				
	Week 1	6.8	2.4–19.0	[9]	Lognormal μ = ln(6.76), σ = 0.52
	Week 2 and 3	3.5	1.3– 8.9		Lognormal μ = ln(3.45), σ = 0.48
	Vaccine-induced RR of IS post dose two during				
	Week 1	2.8	1.1– 7.3	[9]	Lognormal μ = ln(2.84), σ = 0.47
	Week 2 and 3	2.1	1.0– 4.6		Lognormal μ = ln(2.11), σ = 0.39

IS: intussusception; RR: relative risk; RVGE: rotavirus gastroenteritis; SD: standard deviation.

^a^ EGB (Echantillon Généraliste des Bénéficiaires): extraction of acute gastroenteritis admissions in infant before 5 years of age from the French hospital stays database.

**Table 2:** Model input parameters for RotaTeq and their distributions for interval estimation

Input parameter		Mean (n or %)	(SD) or 95% confidence interval	Reference	Probability distribution
Benefit: prevented hospitalisation for RVGE before 5 years of age	Annual incidence of RVGE hospitalisations per 1,000 infants	3.1	(0.4)	^a^	Gamma shape = 61.2, scale = 5.09 e-5
	Number of RVGE deaths per year	16	15–18	[14]	Lognormal μ = ln(16), σ = 0.046
	Efficacy after first dose				
	During first year	58.9%	51.7%–65.0%	[27]^b^	Beta α = 123.5, β = 86.2
	After second year	53.6%	46.4%–59.7%		Beta α = 115.5, β = 99.9
	Efficacy after second dose				
	During first year	77.4%	71.1%–82.1%	[27]^b, c^	Beta α = 170.7, β = 49.8
	After second year	72.1%	65.8%–76.8%		Beta α = 183.0, β = 70.8
	Efficacy after third dose				
	During first year	95.8%	90.5%–98.2%	[27]^b^	Beta α = 99.0, β = 4.34
	After second year	88.0%	82.7%–90.4%		Beta α = 240, β = 32.7
Risk: vaccine-induced hospitalisation for IS before 1 year of age	Annual incidence of IS hospitalisations per 100,000 infants under 1 year of age	28.0	(1.9)	[21]	Gamma shape = 214.4, scale = 1.31e-6
	Case–fatality rate of IS	0.0012	(0.0007)	[32]	Beta α = 3, β = 2584
	Vaccine-induced RR of IS post dose one during				
	Week 1	9.9	3.7–26.4	[9]	Lognormal μ = ln(9.89), σ = 0.49
	Week 2 and 3	6.3	2.8–14.4		Lognormal μ = ln(6.32), σ = 0.41
	Vaccine-induced RR of IS post dose two during				
	Week 1	2.8	1.2–6.8	[9]	Lognormal μ = ln(2.81), σ = 0.44
	Week 2 and 3	1.8	0.8–3.9		Lognormal μ = ln(1.77), σ = 0.39

IS: intussusception; RR: relative risk; RVGE, rotavirus gastroenteritis; SD: standard deviation.

^a^ EGB (Echantillon Généraliste des Bénéficiaires): extraction of acute gastroenteritis admissions in infant before 5 years of age from the French hospital stays database.

^b^ The waning effects between first- and second-year efficacies were considered similar to Rotarix (i.e. a 5.3% decrease after first and second dose, and a 7.8% decrease after third dose [28])

^c^ Efficacy after second dose and its confidence interval were taken as average values between post-dose one and post-dose three results.

### Risk input parameters

The incidence of intussusception hospitalisations was 0.3‰ in 2011 [21], which led to an average of 214 intussusception cases annually. The 80 cases that met level 1 in the Brighton classification and occurred in 2008 and 2009 were selected to fit the age distribution that is displayed on Figure 1 (B). The mortality rate due to intussusception was estimated by three deaths among 2,588 cases, leading to an estimate of 1.1‰ [32]. The lower part of Table 1 summarises the chosen distributions and parameters for Rotarix while Table 2 shows the corresponding information for RotaTeq.

### Benefit–risk estimate

With the current vaccine coverage of 9.5%, rotavirus vaccination is estimated to prevent 1,074 cases of RVGE annually (2.5th–97.5th: 810–1,378) and to induce 5.0 cases of intussusception (2.5th–97.5th: 3.2–7.7) (Table 3).

**Table 3:** Estimated annual benefits and risks of rotavirus vaccine, France, 2015

Vaccine coverage scenario	Event	Benefit (prevented RVGE)		Risk (vaccine-induced IS)		Benefit–risk ratio	
		Median	2.5th–97.5th percentiles	Median	2.5th–97.5th percentiles	Median	2.5th–97.5th percentiles
Current (Rotarix coverage of 6.7% and RotaTeq coverage of 2.9%)	Hospitalisation	1,074	810–1,378	5.0	3.2– 7.7	214	128–362
	Death	1.4	1.2–1. 6	0.005	0.001–0.015	273	89–1,228
Rotarix coverage reaching 92%	Hospitalisation	10,459	7,702–13,498	47.0	25.1–81.4	221	118–437
	Death	13.7	11.1–15.2	0.05	0.01–0.15	284	86–1,339
RotaTeq coverage reaching 92%	Hospitalisation	10,291	7,901–13,087	49.9	28.7–81.8	206	118–376
	Death	13.3	12.1–14.7	0.05	0.01–0.16	263	84–1,180

IS: intussusception; RVGE: rotavirus gastroenteritis.

**Figure 2:**
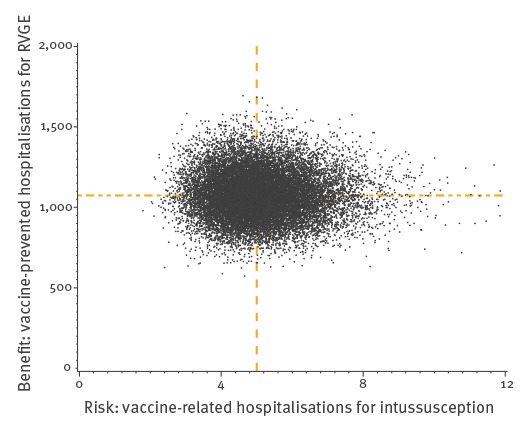
Number of vaccine-prevented hospitalisations for rotavirus gastroenteritis in infants under 5 years of age (benefit) vs vaccine-related hospitalisations for intussusception in infants under 1 year of age (risk) after 20,000 simulations using 2015 estimated vaccine coverage^a^, France

The benefit-risk ratio is one hospitalised intussusception for every 214 (2.5th–97.5th: 128–362) RVGE hospitalisations. Vaccination is also estimated to prevent 1.4 (2.5th–97.5th: 1.2–1.6) RVGE-related deaths annually and to be responsible for 0.005 (2.5th–97.5th: 0.001–0.015) vaccine-related intussusception death, which means that 273 (2.5th–97.5th: 89–1,228) RVGE-related deaths could be prevented for each additional intussusception death. The results of the 20,000 simulations are displayed in Figure 2.

RVGE: rotavirus gastroenteritis.

^a^ In 2015, Rotavirus vaccine coverage was estimated 9.5%.

The dotted lines display the median benefit and risk values.

**Figure 3:**
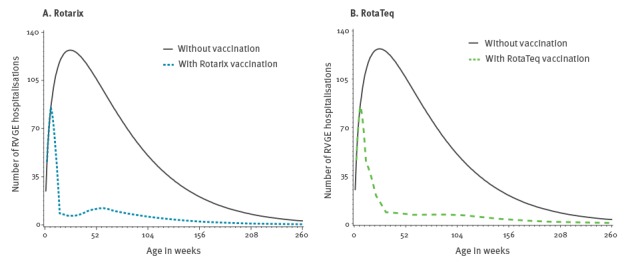
Number of rotavirus gastroenteritis hospitalisations in infants under 5 years of age by vaccination and vaccine type^a^, France, 2015

**Figure 4:**
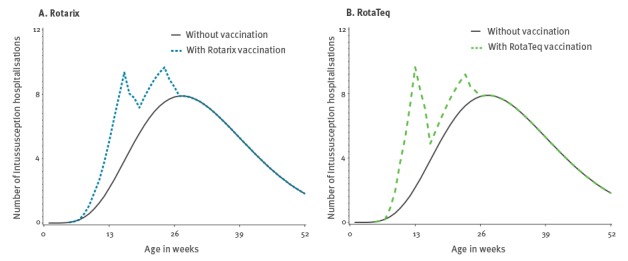
Number of intussusception hospitalisations in infants under 1 year of age by vaccination and vaccine type^a^, France, 2015

^a^ At vaccination coverage of 92%.

With a 92% rotavirus vaccination coverage, ca 3,650,000 children under 5 years of age would be vaccinated. If Rotarix was the only available vaccine, the annual number of prevented RVGE hospitalisations and the annual number of vaccine-induced hospitalisations for intussusception would be 10,459 and 47, respectively. Furthermore, 13.7 RVGE-associated deaths would be prevented and 0.05 vaccine-related intussusception death would be induced. Similar results would be observed if RotaTeq were to be the only available vaccine (Table 3). As shown in Table 3, benefit and risk estimates (either for hospitalisations or death) are less precise with larger vaccine coverage because the dispersion of benefit and risk values is proportional to vaccine coverage; however, the BR ratio is unaffected by changes in vaccine coverage. Figure 3 displays the baseline and prevented number of RVGE hospitalisations while Figure 4 displays the baseline and excess number of intussusception according to each vaccine at a coverage of 92%.

RVGE: rotavirus gastroenteritis.

^a^ At vaccination coverage of 92%.

The influence of the decrease in vaccination efficacy with time was assessed by assuming, in a sensitivity analysis, that efficacy remained constant with time and equal to its highest value. With the 2015 estimated vaccine coverage of 9.5%, the median numbers of prevented hospitalisations and deaths for RVGE increases slightly from 1,074 to 1,121 and from 1.4 to 1.5, respectively.

## Discussion

We show that the BR ratio of rotavirus vaccination in France is 214 RVGE hospitalisations prevented for every additional intussusception hospitalisation, thereby suggesting that vaccine-prevented hospitalisations for RVGE far outweigh vaccine-induced hospitalisations for intussusceptions. Should the national vaccine coverage reach 92%, either with Rotarix or RotaTeq or a mixture of both, our model suggests that ca 10,400 hospitalisations for RVGE would be prevented and ca 50 cases of intussusception would be induced annually.

Some assumptions were made to derive these estimates. Although the annual incidence of RVGE hospitalisations varies over time, we assumed it was constant and used a single average value. The incidence of hospitalisations for RVGE was estimated from the French hospital discharge part of the EGB database, where vaccinated and unvaccinated cases cannot be distinguished. Thus, the risk of hospitalisation for RVGE is not the risk in an unvaccinated population since ca 9.5% of the population is vaccinated. Assuming 100% efficacy, the observed cases occurred in the 90.5% unvaccinated fraction of the population. In addition, removing 9.5% of the children because they had been vaccinated would not markedly change the age distribution of the RVGE cases. To model vaccine coverage in the absence of information on the actual ages at vaccine administration, we assumed a constant vaccination rate within the ages recommended for each dose. Moreover, we also chose an incomplete vaccination rate of 4% as in an earlier Belgian study [18], and assumed that French and Belgian parents behave similarly. This extrapolation could induce either an under- or an over-estimation. Regarding hospitalisations for RVGE, they were defined by the ICD10 code A080 only because there are no microbiological results available in the EGB database. Moreover, duplicates are possible but unlikely because in France, young children should be admitted to paediatric and not to general hospitals. Finally, the chosen vaccine-induced intussusception risk values were those of Carlin's work [9]. For Rotarix, they are higher than the pooled values calculated by Stowe in a meta-analysis of six studies [35]: 6.76 vs 6.03 in the first week after dose one and 2.11 vs 1.83 in the first week after dose two. Choosing smaller values would have increased the BR ratio estimate.

The present model could be complicated in several ways. In each iteration, parameters were drawn from independent distributions although some correlation could have been present, for instance, that in a given child, the vaccine-induced RR of intussusception after the second or third dose would be positively correlated with the RR after the first dose. In addition, although there is herd immunity [36], we did not take this indirect benefit of rotavirus vaccination into account. Consequently, the benefit and BR ratio might be somewhat underestimated.

An overview of results from BR studies in other countries can be found in the third table of Ledent's paper [11] and are briefly recalled here for the purpose of comparison. Our work shows that the BR ratio of 214 for hospitalisations at current estimated coverage of 9.5% (BR ratio = 214) is somewhat lower than those of other reports, e.g. BR ratios range from 282 in Mexico to 1,265 in Brazil. When death is the event of interest, the BR ratio of 273 found in our study is of a similar order of magnitude as those observed in Brazil (BR ratio = 213), Mexico (BR ratio = 331), Japan (BR ratio = 366) and Latin America (BR ratio = 395).

Our findings suggest that a coverage of the French population with 92% rotavirus vaccine would avoid more than 10,000 hospitalisations for RVGE in children under 5 years of age annually, outweighing the intussusception risk by a factor of more than 200. The BR balance for deaths could possibly be even further improved if parents were aware that they should seek medical attention rapidly on the first symptoms after vaccine administration.

The estimated benefits of vaccination in our study greatly exceed the estimated risks and our results should contribute to provide further evidence for discussions around rotavirus vaccination in France.
